# Vaccination with dengue virus-like particles induces humoral and cellular immune responses in mice

**DOI:** 10.1186/1743-422X-8-333

**Published:** 2011-06-30

**Authors:** Shuo Zhang, Mifang Liang, Wen Gu, Chuan Li, Fang Miao, Xiaofang Wang, Cong Jin, Li Zhang, Fushun Zhang, Quanfu Zhang, Lifang Jiang, Mengfeng Li, Dexin Li

**Affiliations:** 1 State Key Laboratory for Molecular Virology and Genetic Engineering, Institute for Viral Disease Control and Prevention, China CDC, 155 Chang Bai Road, Chang Ping District, Beijing 102206, China; 2 Department of Microbiology, Zhongshan School of Medicine, Sun Yat-Sen University, Guangzhou 510080, Guangdong, China

**Keywords:** Dengue virus, VLP, Vaccine

## Abstract

**Background:**

The incidence of dengue, an infectious disease caused by dengue virus (DENV), has dramatically increased around the world in recent decades and is becoming a severe public health threat. However, there is currently no specific treatment for dengue fever, and licensed vaccine against dengue is not available. Vaccination with virus-like particles (VLPs) has shown considerable promise for many viral diseases, but the effect of DENV VLPs to induce specific immune responses has not been adequately investigated.

**Results:**

By optimizing the expression plasmids, recombinant VLPs of four antigenically different DENV serotypes DENV1-4 were successfully produced in 293T cells. The vaccination effect of dengue VLPs in mice showed that monovalent VLPs of each serotype stimulated specific IgG responses and potent neutralizing antibodies against homotypic virus. Tetravalent VLPs efficiently enhanced specific IgG and neutralizing antibodies against all four serotypes of DENV. Moreover, vaccination with monovalent or tetravalent VLPs resulted in the induction of specific cytotoxic T cell responses.

**Conclusions:**

Mammalian cell expressed dengue VLPs are capable to induce VLP-specific humoral and cellular immune responses in mice, and being a promising subunit vaccine candidate for prevention of dengue virus infection.

## Background

Dengue viruses (DENV) are transmitted among humans by mosquitos, such as *Aedes aegypti *and *Aedes albopictus *[1]. DENV infection may cause a self-limited febrile illness known as dengue fever (DF), or result in a life-threatening dengue hemorrhagic fever or dengue shock syndrome (DHF/DSS). It has been estimated that 50-100 million cases of DF and 250,000-500,000 cases of DHF occur annually [2], mainly in tropical and subtropical regions of the world. Dengue viruses, exist as four serotypes, belong to the family of *Flaviviridae*, genus *Flavivirus*. The virion contains a positive-sense single-strand RNA genome with a long open reading frame coding for capsid (C), premembrane(prM), and envelope(E) structural proteins, as well as seven non-structural(NS) proteins: NS1, NS2A, NS2B, NS3, NS4A, NS4B, and NS5[3].

Because of the widespread geographical distribution and the severe clinical symptoms, dengue vaccine is urgently needed. However, licensed vaccine is not currently available for prevention of DENV infection. One major reason is the phenomenon of antibody dependent-enhancement (ADE), which is known as that a subsequent infection with an alternate serotype can enhance severity of dengue disease [1]. One explanation of this phenomenon is that pre-existing non-neutralizing antibodies may enhance capacity of the new infecting DENV to access FcγR bearing cells. Therefore, DENV infection commonly lacks of antibody cross-protection among serotypes. Various strategies have been used to develop dengue vaccine. The most promising candidates are the live-attenuated tetravalent vaccines of which the clinical trials are in progress [4-7]. One example is the Sanofi Pasteur's dengue vaccine candidate, which is based on a backbone of yellow fever vaccine (YF 17D) replication genes and incorporates the envelope genes of the four dengue virus serotypes, entered its final stage of clinical development in Australia. However, concerns have been raised about interference in virus replication among serotypes [8]. If the replication of four serotypes of vaccine viruses is not balanced, the replication of non-dominant serotypes can be interfered by dominant serotypes, which can result in preferential antibody response to the dominant strains and lead to a risk of developing more serious disease [9]. Thus, an ideal dengue vaccine should induce neutralizing antibody responses against all four serotypes simultaneously and it must be safe to use.

To develop an effective and safe dengue vaccine, we tested the effect of recombinant dengue virus-like particles (VLPs). Virus-like particle vaccine has shown considerable promise as vaccine candidate for many viral diseases [10-13]. VLPs, which are similar to infectious virions in the structural and physicochemical features, are non-infectious particles and have advantages in safety and manufacturing. VLPs can be produced in multiple expression systems such as E.coli, yeast, baculovirus and mammalian cells. Recombinant VLPs can be efficiently taken up, internalized and processed by antigen presenting cells (APCs) [11], and capable to elicit strong humoral and cellular immune responses against viruses [14-16]. Recombinant VLPs of flaviviruses have been shown to be produced efficiently by co-expressing the prM and E proteins in the absence of C protein [17-19].

In this study, four serotypes of dengue virus-like particles containing recombinant prM and E proteins were generated in mammalian cells, and their immunogenicity was evaluated in BALB/c mice. The results showed that monovalent VLPs of each serotype could stimulate specific IgG and neutralizing antibody against homotypic virus, and tetravalent VLPs could induce specific IgG and neutralizing antibodies against all four serotypes of dengue virus. Moreover, vaccination with monovalent or tetravalent VLPs also resulted in the induction of specific cellular responses. Therefore, dengue VLPs can be a potential vaccine candidate for the prevention of dengue infection.

## Materials and methods

### Cells and viruses

293T cells (ATCC No.CRL-11268) were cultured in Dulbecco's Modified Eagle Medium (DMEM; Gibco) supplemented with 10% heat-inactivated fetal bovine serum (FBS), penicillin (100 U/ml) and streptomycin (100 μg/ml) at 37°C with 5% CO_2. _C6/36 *Aedes albopictus *cells (ATCC No.CRL-1660) were grown at 28°C without CO_2 _in Eagle's Minimum Essential Medium (EMEM; Gibco) supplemented with FBS, penicillin and streptomycin as well.

Each serotype of dengue virus was passaged and propagated in C6/36 cells. The DENV-1 strain GZ01/95 and DENV-2 strain ZS01/01 were supplied by the Department of Microbiology, Zhongshan School of Medicine, Sun Yat-sen University, China. DENV-1 strain Hawaii, DENV-2 strain NGC, DENV-3 strain H87 and DENV-4 strain H241 were preserved by our laboratory. Strain GZ01/95, ZS01/01, H87 and H241 were used for RNA extraction and then VLPs expression plasmids construction while strain Hawaii, NGC, H87 and H241 were used for neutralization analysis. Japanese encephalitis virus (JEV) strain SA_14_-14-2 was also propagated in C6/36 cells and mainly used for cDNA cloning.

### Construction of DENV VLP expression plasmids

The QIAamp Viral RNA Kit (Qiagen, Santa Clarita, CA) was used to extract genomic RNA of DENV1-4 and JEV from 140 μl C6/36 cells culture supernatant infected with each virus. The extracted RNA was subjected to RT using Transcriptor High Fidelity cDNA Synthesis Kit (Roche, Cat. No. 05081955001) to generate cDNA templates for amplification of target genes. The PCR segments were digested with NheI and NotI enzymes and inserted into NheI and NotI sites of pcDNA5/FRT vector.

**Figure 1:**
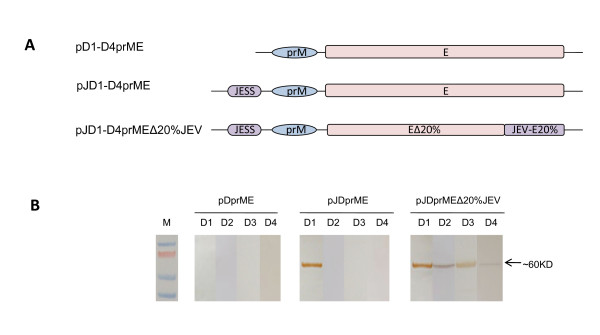
**Construction strategies and secretion analysis of DENV1-4 VLPs**. **A) **Schematic representation of three strategies to construct DENV1-4 VLP expression plasmids, pD1-D4prME include the entire prM and E gene regions of DENV. JEV signal sequence (JESS) is introduced to the N-terminal of pJD1-D4prME, and pJD1-D4prMEΔ20%JEV. The corresponding JEV-E sequence was constructed in pJD1-D4prMEΔ20%JEV to replace the 20% C-terminal region of DENV-E; **B) **Western blot analysis of DENV1-4 VLPs secretion, Concentrated culture supernatants from 293T cells transformed with pD1-D4prME, pJD1-D4prME or pJD1-D4prMEΔ20%JEV were tested by rabbit polyclonal serum specific against dengue E protein. Bound antibodies were detected by a HRP-conjugated goat anti-mouse antibody. The band corresponding to envelope (E) is indicated with an arrow to the right of the blot.

Three types of expression plasmids were constructed for each serotype of DENV (Figure 1A). The first type of plasmids was named pD1-D4prME, with entire prM and E genes of each DENV serotype cloned into the pcDNA5/FRT vector. The second type of plasmids was pJD1-D4prME, with entire prM and E genes of each DENV serotype and an optimized JEV signal sequence(JESS) [17,20,21] gene from SA_14_-14-2 strain. To further compare the impact of E protein transmembrane and cytoplasmatic domains, the third type of plasmids was designed as chimeric constructs pJD1-D4prMEΔ20%JEV, which contained JESS and full length of prM, but replaced the 3' terminal 20% region of E gene with the corresponding sequence of JEV (strain SA_14_-14-2).

### Transient transfection of 293T cells with DENV VLP expression plasmids

293T cells were prepared in wells of 6-well plates one day earlier and were transfected with pD1-D4prME, pJD1-D4prME or pJD1-D4prMEΔ20%JEV for each well using lipofectamin2000 (Invitrogen, Cat no.11668019) according to instructions supplied by the manufacturer. Briefly, for each plasmid transfection, 8 μl lipofectamin2000 was diluted in 200 μl Opti-MEM (Gibco), and after incubating for 5 min at room temperature the diluted lipofectamin2000 was combined with diluted plasmid DNA (4 μg diluted in 200 μl Opti-MEM). After 20 min's incubation at room temperature, the mixture was added to each well with 80%-90% confluence of 293T cells. 48 hours post-transfection, cells and supernatants were harvested for future use.

### Western blot analysis

Concentrated culture supernatants were applied to a NuPAGE 4-12% Bis-Tris gradient gel, and followed by electroblotting onto PVDF membrane. Non-denatured proteins were then probed with E protein specific rabbit polyclonal sera which were produced in our laboratory. A goat anti-rabbit IgG conjugated to HRP was used as the secondary antibody. The reactions were detected by 3,3' diaminobenzidine (DAB) reagent according to the manufacturer's instructions.

### Purification of DENV VLPs and virions

For DENV1-4 VLPs purification, 293T cells in T175 flasks were transiently transfected with optimized plasmids of each serotype. The culture supernatants containing extracellular VLPs were harvested routinely every 2 days, continuously repeated 3 times. To purify DENV1-4 virions which were used for electron microscopy and mice immunization, C6/36 cells in T175 flasks were infected with DENV strain Hawaii, NGC, H87 and H241 respectively. At 7 days after infection, the supernatants were harvested and inactivated with 1:2000 β-propionolactone.

Cell supernatants collected from dengue VLPs production cells or dengue virus infected C6/36 cells were clarified by centrifugation at 10 000× g for 30 min followed by concentration using ultrafiltration system Vivaflow 200 (Sartorius stedim biotech). The concentrated culture supernatants were then purified by a two-step ultracentrifugation. The first step was a rate zonal centrifugation. Samples were added on the top of a 15-60% sucrose gradient and ultracentrifuged in a SW41 rotor (Beckman Coulter Inc.) at 38,000 rpm at 4 °C for 4 h. About 1 ml fractions were collected and pelleted by a second ultracentrifugation and then resuspended in PBS. The total protein concentrations of authentic virions and dengue VLPs were determined by Pierce BCA Protein Assay Kit.

### Transmission Electron microscopy (TEM)

Purified dengue VLPs and dengue virions from sucrose density-gradient fractions were fixed with 2% glutaraldehyde. Small droplets of fixed samples were placed on copper formvar-coated grids for 1 min, then the grids were stained with sodium phosphotungstate for 1 min (excess samples of each step were removed). At last, the grids were visualized by TEM.

### Mice immunization

Four to six-week-old female BALB/c mice were purchased from Chinese Academy of Medical Sciences Breeding Laboratories and were intraperitoneally (i.p.) inoculated with monovalent DENV VLPs (100 μg per dose) or a tetravalent combination (25 μg of each serotype per dose) in Freund's complete adjuvant (Sigma) for priming and in Freund's incomplete adjuvant for two times of boosting at an interval of 2 weeks. Equal amount of DENV virions (100 μg for monovalent vaccine and 25 μg of each serotype for tetravalent vaccine) were used as controls with the same regimen. On days 0, 14 and 28, blood samples were collected through tail vein for measurement of serum IgG. At 2 weeks after the last inoculation, mice were sacrificed to collect serum for the neutralizing antibodies assay and separated splenocytes for testing cytotoxic T cell responses.

### ELISA to measure serum IgG

VLPs specific serum IgG antibodies were titred by the binding capacity with rEIII protein, a recombinant protein that chimericly expressed DENV1-4 EIII domains in a certain order and previously produced in our laboratory. IgG titers were measured using enzyme-linked immunosorbent assay (ELISA). Briefly, 200 ng purified rEIII per well was coated on 96-well plates at 4°C overnight. Then, the plates were blocked with 5% skimmed milk in PBS for 1 h, and incubated with 2-fold serial diluted serum samples (starting from 1:50) at 37°C for 1 h. Bound IgG was detected by HRP-conjugated goat anti-mouse IgG (Sigma). After addition of 3,3', 3,5'-tetramethylbenzidine (TMB), absorbance was measured at 450 nm. The value which exceeds the mean+2 S.D. of negative control was considered positive.

### Antibody neutralization assay

The neutralization ability of serum antibodies against DENV was determined using CPE-determination assays. Briefly, mice sera from all groups were heat-inactivated at 56°C for 30 min, then the sera were two-fold serial diluted from 1:5 to 1:160 in Eagle's medium supplemented with 1% heat-inactivated FBS, penicillin and streptomycin and mixed with 100TCID50 virus. After 1 h incubation at 37°C, 100 μl of virus-serum mixture was inoculated to the confluent monolayer of BHK-21 cells in 96-well plates. Every dilution of each serum was performed in quadruplicate. The plates were then incubated in a CO_2 _incubator at 37°C for 7 days. The neutralization titer was expressed as the maximum serum dilution at which the CPE of the virus was not observed in all four wells.

### Enzyme Linked Immunospot (ELISPOT) Assay

The ELISPOT 96-well plates (BD) were coated with 100 μl of anti-mouse IFN-γ (5 μg/ml in coating buffer) at 4°C overnight. The following day, plates were washed and blocked with blocking solution for 2 h. Then, 100 μl freshly isolated splenocytes (5 × 10^5 ^cells) from the immunized mice were added to each well and stimulated with DENV VLPs at 37°C for 40 h. After cells were washed out, biotinylated anti-mouse IFN-γ was added to each well and incubated for 2 h at room temperature. Thereafter, the plates were washed and incubated for 1 h at room temperature with streptavidin-HRP. Finally, AEC substrate solution (BD) was added and spots were counted by ImmunoSpot^® ^Analyzer (Cellular Technology Ltd.).

### Statistical analysis

Statistical comparisons among groups were analyzed by one way ANOVA using SPSS 11.5. A P value less than 0.05 was considered statistically significant.

## Results

### Production of DENV VLPs

To optimize the production of DENV VLPs, three types of expression plasmids encoding prM and E glycoproteins were constructed for each serotype (Figure 1A). E and prM proteins were chosen as two subunits of recombinant VLPs, because the former one constitutes the spikes on DENV membrane surface and is known as the major protective antigen of DENV to induce neutralizing antibodies, and the latter one is also embedded in the viral envelop and contributes to the stability of E protein. To test the secretion of DENV VLPs from transient transfected 293T cells, culture supernatants of 293T cells were collected and identified by western blot analysis for E protein expression (Figure 1B). Cells transfected with pD1-D4prME plasmids that express full length of prM and E proteins could express intracellular proteins (data not shown). However, due to the lack of signal sequence, they could not secret VLPs into tissue culture. When adding a JEV signal sequence at the N-terminal of full length of prM and E genes, cells transfected with expression plasmid pJD1prME could effectively secret VLPs, but cells transfected with pJD2-D4prME still could not secret VLPs. When both carrying a N-terminal JEV signal sequence and replacing C-terminal 20% regions of DENV E gene with the corresponding region of JEV E gene, all four constructs, pJD1-D4prMEΔ20%JEV, could secrete VLPs into tissue culture. Therefore, we chose pJD1prME and pJD2-D4prMEΔ20%JEV to express recombinant DENV1 and DENV2-4 VLPs in 293T cells, respectively.

**Figure 2:**
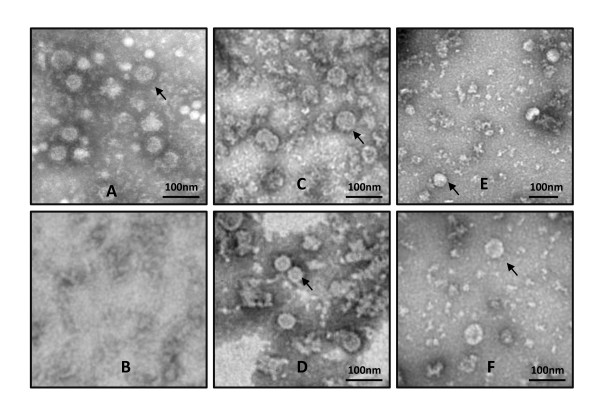
**Morphology and size of dengue VLPs and virions**. Purified dengue virions (arrow indicated in **A**) and VLPs (arrow indicated in **C-F**) were negatively stained and analyzed by TEM. Scale bar indicates 100 nm. A. Dengue virions, B. Negative control, C. DENV-1 VLPs, D. DENV-2 VLPs, E. DENV-3 VLPs, F. DENV-4 VLPs.

To compare the size and morphology of recombinant DENV1-4 VLPs and their corresponding serotype of DENV virions, purified dengue VLPs and virions were observed under transmission electron microscopy (TEM) (Figure 2). All four types of recombinant VLPs exhibited as electron-dense spherical particles of 45-55 nm size, which were similar to the morphology and size of DENV virions. Therefore, by optimizing the express plasmids and using mammalian 293T cells, we successfully acquired DENV VLPs which consisted of major antigenic proteins of the virus and exhibited similar morphological features as nature virus particles.

### DENV VLPs elicited virus specific IgG and neutralizing antibodies

**Figure 3:**
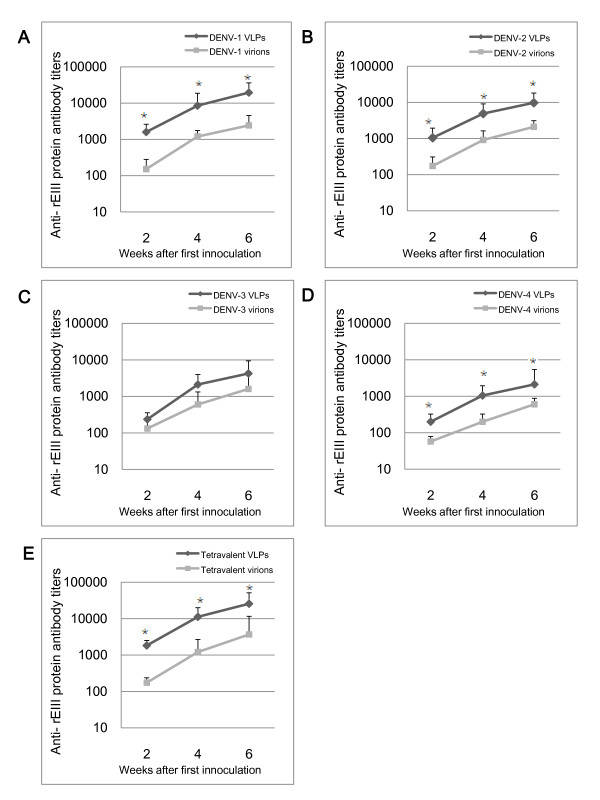
**Virus specific IgG were enhanced by DENV VLPs or virions**. BALB/c mice were intraperitoneally immunized with 100 μg monovalent DENV VLPs or virions (**A-D**), or tetravalent VLPs or virions (**E **25 μg of each serotype) for three times at two-week intervals. At day 14, 28, 42, sera were collected and ELISA was used to test for rEIII specific IgG. Data were expressed as mean titer with a standard deviation (SD) bar. *indicates statistical significance (P < 0.05).

**Figure 4:**
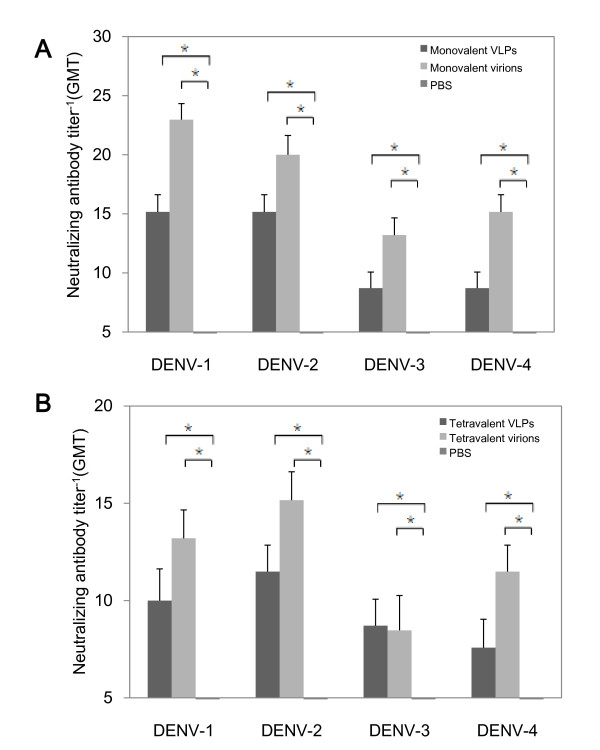
**Serum neutralizing antibody titer of vaccinated mice**. BalB/C mice were immunized with **A) **100 μg monovalent VLPs or virions, **B) **tetravalent VLPs or virions (25 μg of each serotype) for three times at two weeks interval. At day 42, the serum neutralizing antibodies were assessed using CPE-determination assays. Data of each group was expressed as geometric mean titer (GMT) with an S.D. bar (n = 5). *indicates statistical significance (P < 0.05).

To evaluate humoral responses induced by recombinant VLPs, BALB/c mice were immunized with monovalent dengue VLPs of each serotype or their corresponding virion counterparts for three times at two-week intervals. Serum samples were collected after 2 weeks of each immunization, and analyzed for IgG antibodies specific against rEIII, a recombinant protein that expressed chimerical EIII domains of all four DENV serotypes. EIII domain of DENV contains several neutralizing epitopes and host cell receptor recognition sites. As shown in Figure 3A-D, in comparison to the PBS control, mice immunized with either dengue VLPs or virions induced high level of rEIII specific serum IgG. For DENV-1, DENV-2 and DENV-4 VLPs, they induced even higher IgG responses than DENV-1, DENV-2 and DENV-4 virions, respectively. Neutralization assays using serum collected on day 42 demonstrated that DENV1-4 VLPs could elicit comparable level of homotypic neutralizing antibodies as monovalent dengue virions (Figure 4A).

The results that monovalent dengue VLPs could efficiently enhance specific IgG and develop neutralization antibodies, suggested that the tetravalent formulation of DENV VLPs, which was constituting of four types of DENV VLPs at equal amount, might be capable to stimulate neutralizing antibodies against all four serotypes of DENV. As generating balanced neutralizing antibodies to each serotype of a tetravalent dengue vaccine is desired for its safety and efficacy, a tetravalent dengue VLPs combination was applied to BALB/c mice for all priming and boosting immunizations as the regimen used for monovalent VLPs vaccination. Tetravalent dengue virions and PBS were again used as controls. The serum samples collected at Day 14, 28, and 42 post the initial tetravalent VLPs vaccination, were analyzed for rEIII specific IgG antibodies. The data showed that tetravalent virions could stimulate IgG antibodies against rEIII protein. Intriguingly, tetravalent VLPs could even achieved to a higher level than tetravalent virions could induce (Figure 3E). Moreover, neutralizing antibodies stimulated by tetravalent VLPs exhibited simultaneous blocking of DENV1-4 infections, and the blocking effect was comparable to tetravalent formula of virions (Figure 4B).

Therefore, similar as DENV virions, both monovalent and tetravalent formula of DENV VLPs could effectively induce virus specific IgG and produce high titer of protective neutralizing antibodies in vaccinated mice.

### DENV VLPs induced virus-specific T cell responses

**Figure 5:**
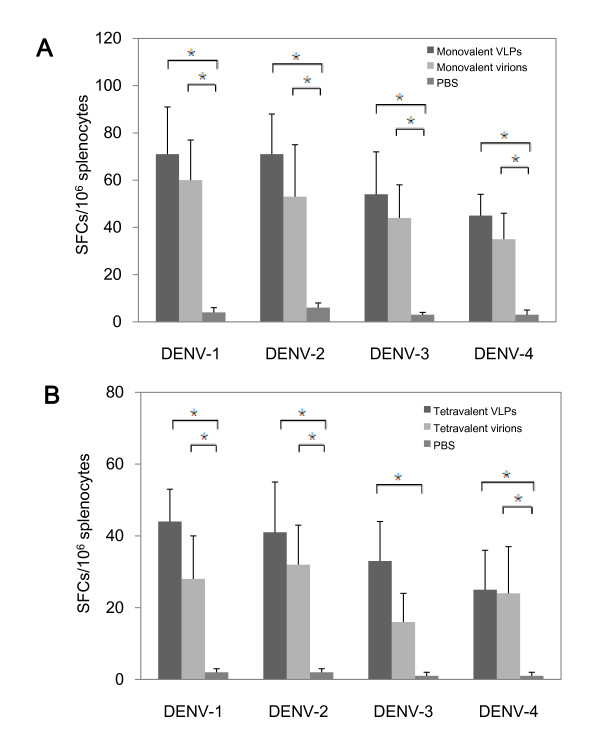
**Dengue specific T cell responses evaluated by ELISPOT assay**. BALB/c mice were immunized with DENV1-4 VLPs or virions in either monovalent **A) **or tetravalent **B) **formula. The immunized mice were sacrificed at day 42 and the collected spleen cells were isolated and stimulated *in vitro *with each of DENV1-4 VLPs. ELISPOT assay was performed to test IFN-γ production. The mean number of spot forming cells (SFCs)/10^6 ^splenocytes was shown as VLPs-stimulated plus mock-stimulated with an S.D bar. *indicates statistical significance (P < 0.05).

Lastly, to investigate cellular immune responses triggered by dengue VLPs, ELISPOT assay was employed to test dengue specific cytotoxic T cell responses. At day 42 after the initial immunization, mice were sacrificed and spleen cells were collected. Each of the monovalent DENV1-4 VLPs immunized spleen cells were then *in vitro *stimulated with corresponding DENV1-4 VLPs and monitored for IFN-γ production. As depicted in Figure 5A, splenocytes from mice immunized with dengue monovalent VLPs exibited specific cytotoxic T cell responses, and the extent was similar to the effect of monovalent DENV virions. Furthermore, dengue tetravalent VLPs manifested high level of IFN-γ to *in vitro *stimulation with each serotype of DENV1-4 VLPs, and with no significant differences to the homotypic virions (Figure 5B). These results demonstrated that vaccination with either monovalent or tetravalent dengue VLPs could elicit dengue specific cellular immune responses.

## Discussion

Despite many years of efforts, an effective dengue vaccine has not been developed. Various strategies have been applied to develop dengue vaccine, such as attenuated [22,23], subunit [24,25], chimeric [26,27], and DNA [1,28] vaccines. Previous studies have shown that co-expressed prM and E proteins of dengue virus could produce VLPs in mammalian cells, but the VLPs expression plasmids were often used as DNA vaccine [17,20,29]. Here we studied on recombinant dengue VLP vaccine as a new candidate. VLPs are similar to infectious virions in both structural and biochemical properties but are non-replicating and free of genome. Therefore, due to their preserved immunogenicity in native forms and better safety, VLPs have been used in many vaccine researches for prevention of viral diseases [11].

In order to optimize the production of dengue VLPs, three types of expression plasmids were constructed. The indirect fluorescent antibody (IFA) assay showed all constructs could express intracellular VLPs (data not shown). However, the western blot analysis of culturing supernatant showed that, pD1-D4prME constructs without signal peptide could not secret dengue VLPs. And except for pJD1prME, the constructs simply adding a JEV signal sequence could not secret dengue VLPs. All constructs which replaced the carboxy-terminal 20% of DENV E protein with corresponding JEV E protein could secret VLPs into cell culturing supernatant. Based on data from these express plasmids, pJD1prME and pJD2-D4prMEΔ20%JEV were identified as optimized VLP formation constructs for each DENV serotype. These data from different express plasmids indicated that i) signal peptide was one of the most important factors that influence downstream protein translocation and topology, thus dictating correct processing of dengue virus prM and E proteins by the host encoded signalase and endopeptidase [20]. ii) the transmembrane domain of dengue E protein contains a strong ER retention signal [30,31], replacement of the carboxy-terminal 20% of DENV E protein with the corresponding region of JEV provides extracellular secretion of DENV2-4 VLPs, but does not produce additional benefit to promote extracellular secretion of DENV-1 VLPs.

This was different from previous studies [29,32], which showed that 20% JEV sequence replacement was absolutely necessary for DENV-1 and DENV-2 VLPs secretion; DENV-3 plasmids containing either the full-length DENV-3 E protein gene or the 20% JEV sequence replacement secreted VLPs to similar levels; Whereas DENV-4 VLPs were secreted to high levels by plasmids containing the full-length DENV-4 E protein gene but not by the chimeric plasmid containing 20% JEV E replacement. Considering that dengue viruses of different serotypes or even among different strains of the same type, their biological characteristics were not the same, thus it is essential to use different strategies when constructed dengue VLPs expression plasmids. As dengue particle assembly and secretion is influenced by interaction of prM and E [29], we inferred that the chimeric E (80%DENV and 20%JEV) of DENV2-4 could interact with DENV2-4 prM, which help to stabilize the interaction between prM and E and lead to efficient secretion of VLPs. As for DENV-1 (strain GZ01/95), its prM and E domains might interact stably enough, and the replacement of 20% C-terminal region of E would not improve more on this interaction, therefore DENV-1 VLPs showed similar secret pattern between pJD1prME and pJD1prMEΔ20%JEV plasmids.

The immunogenicity of dengue VLPs was evaluated using BALB/c mice. The analysis of humoral immune responses revealed that dengue VLPs, in either monovalent or tetravalent formula, induced high levels of rEIII-specific IgG. Because dengue virus-induced neutralizing antibodies can bind to virus and prevent virus from binding to host cell receptors, therefore inducing neutralizing antibodies is particularly important to block virus entry into target cells [33]. In this study, either the monovalent or the tetravalent formula of dengue VLPs could efficiently trigger *in vivo *development of neutralizing antibodies. Furthermore, the tetravalent formula of VLPs was able to simultaneously induce balanced neutralizing antibodies against all four serotypes. All these results confirmed that all four serotypes of DENV VLPs prepared in this study preserved the antigenicity of prM and E proteins.

One of the marked advantages of VLPs is their ability to induce cellular immunity [34,35]. Since IFN-γ constitutes a major mediator of the Th1 cell-mediated immune response and has been shown to play a key role in antiviral activity against dengue [36], in our study, cellular immune responses were assessed by IFN-γ releasing ability of VLPs-stimulated spleen cells. Spleen cells from mice vaccinated with dengue VLPs and virions produced comparable levels of IFN-γ after *in vitro *stimulation with dengue VLPs.

In conclusion, by optimizing the expression plasmids, we successfully generated recombinant DENV1-4 VLPs in mammalian cells. Furthermore, the vaccination effect of VLPs in mice showed that either monovalent or tetravalent formula of dengue VLPs could efficiently elicit virus specific humoral and cellular immune responses. These results supplied evidence that VLP vaccine may serve as a promising strategy for dengue vaccine development.

## Competing interests

The authors declare that they have no competing interests.

## Authors' contributions

ZS performed most of the experiments and involved in manuscript preparation. LM coordinated laboratory manipulation and edited the manuscript. GW participated in mice immunization and detection of humoral immune responses. LC, MF, WX and ZQ were involved in cells culture, virus infection and VLPs purification. JC participated in editing of the manuscript. ZL and ZF participated in the detection of cellular immune responses. JL and LM provided Chinese strains of dengue viruses and gave advices for the project. LD is the project leader and was involved in project design, manipulation, data analysis and finalization of the manuscript. All authors read and approved the final manuscript.
